# The Influence of Organic Vanadium Complexes on an Antioxidant Profile in Adipose Tissue in Wistar Rats

**DOI:** 10.3390/ma15051952

**Published:** 2022-03-06

**Authors:** Renata Francik, Jadwiga Kryczyk-Kozioł, Mirosław Krośniak, Sławomir Francik, Tomasz Hebda, Norbert Pedryc, Adrian Knapczyk, Mehmet Berköz, Zbigniew Ślipek

**Affiliations:** 1 Department of Bioorganic Chemistry, Chair of Organic Chemistry, Jagiellonian University Medical College, Medyczna 9, 30-688 Krakow, Poland; 2 Institute of Health, State Higher Vocational School, Staszica 1, 33-300 Nowy Sącz, Poland; 3 Department of Food Chemistry and Nutrition, Jagiellonian University Medical College, Medyczna 9, 30-688 Krakow, Poland; jadwiga.kryczyk@uj.edu.pl (J.K.-K.); mfkrosni@cyf-kr.edu.pl (M.K.); 4 Department of Mechanical Engineering and Agrophysics, Faculty of Production Engineering and Energetics, University of Agriculture in Krakow, Balicka 120, 30-149 Krakow, Poland; tomasz.hebda@urk.edu.pl (T.H.); norbert.pedryc@urk.edu.pl (N.P.); adrian.knapczyk@urk.edu.pl (A.K.); zbigniew.slipek@urk.edu.pl (Z.Ś.); 5 Department of Biochemistry, Faculty of Pharmacy, Van Yuzuncu Yil University, Van 65090, Turkey; mehmet.berkoz@yahoo.com; 6 Technical Institute, State Higher Vocational School, Staszica 1, 33-300 Nowy Sącz, Poland

**Keywords:** organic vanadium complexes, adipose tissue, antioxidants, high-fat diet, metabolic rate

## Abstract

One of the aspects of biological activity of vanadium is its influence on carbohydrate metabolism. For more than 30 years, various vanadium complexes have been tested as antidiabetic agents. This study researched organic vanadium complexes with bipyridinium ligands and their influences on metabolic rate, as well as on the antioxidant activity of adipose tissue. The effects of sodium (2,2′-bipyridine) oxidobisperoxovanadate (V) octahydrate (known as the V complex), bis(2,2′-bipyridine) oxidovanadium (IV) sulfate dehydrate (known as the B complex), and bis(4.4′-dimethyl-2,2′-bipyridine) oxidovanadium (IV) sulfate dihydrate (labelled as the BM complex) were assessed. Solutions of the tested complexes were introduced intraperitoneally with a probe to animals fed with either a control diet or a high-fat diet. The BM complex had a significant influence on the increase in ferric reducing antioxidant power, as well as on the concentration of glutathione in the adipose tissue of rats fed with a high-fat diet. The V complex increased the concentration of glutathione in the adipose tissue of rats fed with control fodder, as well as significantly reduced the relative change in rat weight for the high-fat diet. Furthermore, the presence of each tested vanadium complex had an impact of statistically significant increase in basal metabolic rate, regardless of applied diet. Further research on these organic vanadium complexes is necessary to understand the mechanisms responsible for their ability to affect adipose tissue.

## 1. Introduction

Nowadays, excess body weight and obesity have become a worldwide problem [1]. Over the last decade, the percentage of the population with an obesity problem has increased from 10% to 40% in most European countries [2]. These dependencies are explained, among others, by disturbance in redox states [3], especially if taken into consideration that antioxidant body capacity inversely correlates with central obesity and body fat [4]. Moreover, a diet rich in fats and carbohydrates may induce more severe oxidative stress and inflammation in obese people in comparison to those with a healthy BMI (Body Mass Index) [5].

At present, adipose tissue is considered to be an endocrine organ. Adipocytes produce adipocytokines, such as adiponectin, resistin plasminogen activator inhibitor–1, tumor necrosis factor alpha, or interleukin-6 [6]. The profile of biological effects of these molecules is broad and includes, inter alia, regulation of energy expenditure, insulin sensitivity, and fatty acid oxidation [7]. The excess of oxidative stress in adipocytes may influence the disturbance of adipocytokine secretion, which might explain the coexistence of obesity with metabolic syndromes [8]. The intensification of oxidative stress in fatty tissue, in turn, may be associated with various factors, such as cellular damage because of the enormous size of adipocytes [9] or disturbance in mitochondrial metabolism by excessive oxidation of fatty acids [10]. Moreover, adipocytes constitute a source of proinflammatory cytokines that lead to chronic inflammation under obesity conditions [11]. 

For these reasons, the attempt to find new strategies to alleviate oxidative stress in obesity by influencing fatty tissue seems to be justified and, thus, has become the object of many research studies [12,13,14]. Alcalá et al. [12], investigated the influence of vitamin E combined with a high-fat diet on the activity of selected antioxidative enzymes and on the parameters characterizing the level of oxidative stress in adipose tissue. Farhangi et al. [13] and Sohet et al. [14], studied the effects of vitamin D combined with a high-fat diet and CoQ10 combined with a fat-fructose diet on selected parameters of oxidative stress in adipose tissue, respectively. As vanadium complexes also have shown antioxidant activity in rats with streptozotocin-induced diabetes [15,16], they may prove to be an interesting alternative in the case of antioxidative status disturbance in adipose tissue in the context of obesity. 

The evaluation of the influence of vanadium complexes on the parameters of the oxidative state in adipose tissue has not yet been investigated, both with regard to their beneficial health effects in living organisms, as well as to their contributions to pathological changes. Such complexes have been shown to possess insulinomimetic properties, and their efficacy has been relatively low in doses [17,18,19].

Considering the above, we aimed to test the influence of three organic vanadium complexes (sodium [2,2′-bipyridine)oxidobisperoxovanadate (V) octahydrate, bis (2,2′-bipyridine)oxidovanadium (IV) sulfate dehydrate, and bis (4,4′-dimethyl-2,2′-bipyridine)oxidovanadium (IV) sulfate dihydrate) combined with high-fat and control diets on the antioxidant activity in adipose tissue. Nowadays, the prevailing view is that adipose tissue is actively involved in metabolic changes in organisms. Based on our conducted research, we present the effects of vanadium complexes on the antioxidant state in adipose tissue, the importance of this tissue in metabolic changes, and its participation in protection from excessive amounts of free radicals.

## 2. Materials and Methods

All rats were treated according to the “Guide for the Care and Use of Laboratory Animals” of the National Academy of Sciences. All procedures were conducted with the ethical approval of the I Local Ethics Committee for Animal Experiments of Jagiellonian University in Krakow (80/2009 17 September 2009).

Using the MetaSite computer program (v 6.0.3, Molecular Discovery Ltd., Borehamwood, Hertfordshire, UK), which allows for the prediction of xenobiotic metabolism, applicable metabolites were selected for the tested ligands. MetaSite is a computational model that enables the prediction of cytochrome P450 (CYP450) dependent metabolism in phase I biotransformations. This software is primarily designed to indicate the atoms in the molecular structure that are most vulnerable to metabolic changes resulting from the cytochrome action.

### 2.1. Reagents

The reagents were purchased from Sigma Aldrich Chemical Company (Steinheim, Germany) and Avantor Performance Materials (Gliwice, Poland).

### 2.2. Synthesis of Vanadium Complexes

**Figure 1:**
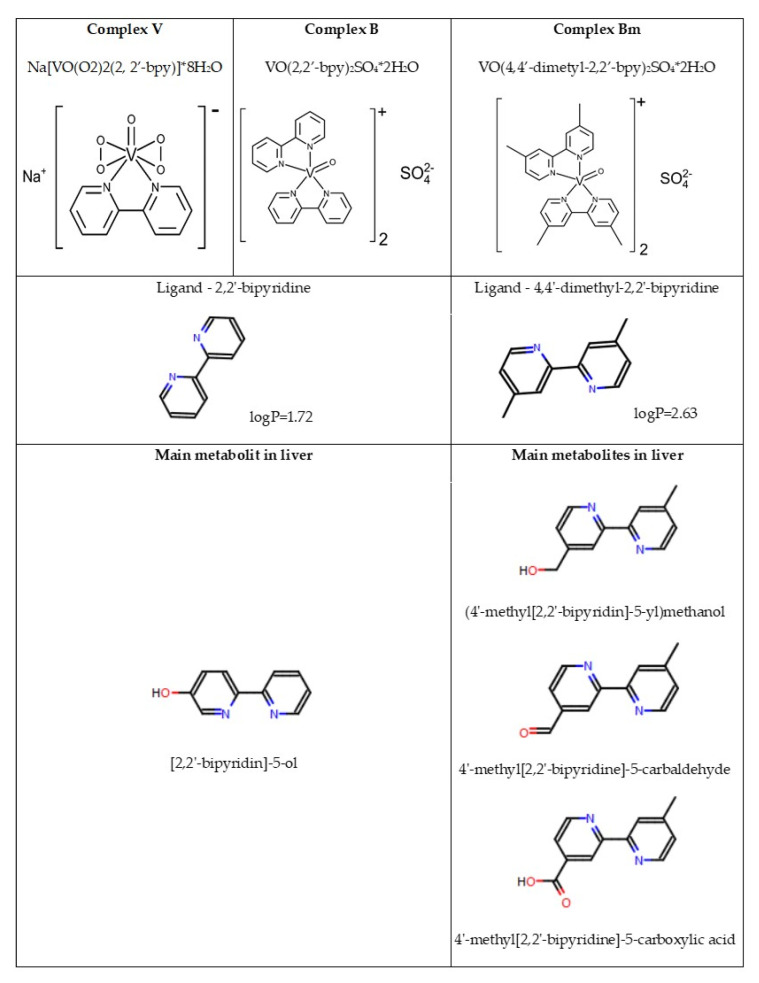
Formulas and structures of the organic vanadium complexes. Metabolites of ligands contained in the complexes based on MetaSite 6: complex V, (2,2′-bipyridine) oxidobisperoxovanadate(V) octahydrate; complex B, bis (2,2′-bipyridine)oxidovanadium(IV) sulfate dehydrate; and complex BM, bis(4,4′-dimethyl-2,2′-bipyridine)oxidovanadium (IV) sulfate dehydrate.

The findings concerning synthesis of the tested vanadium complexes were described in our previous publication [20]. In this study, the following complexes were used: sodium (2,2′-bipyridine)oxidobisperoxidovanadate (V) octahydrate, Na[VO(O_2_)_2_(2,2′-bpy)]⋅8H_2_O, marked as V (453.9 g/mol); bis(2,2′-bipyridine)oxidovanadium (IV) sulfate dihydrate, [VO(SO_4_)(2,2′-bpy)]⋅2H_2_O, marked as B (511.21 g/mol); and bis(4,4′-dimethyl-2,2′-bipyridine)oxidovanadium (IV) sulfate dihydrate, [VO(4,4′-Me-2,2′-bpy)_2_]SO_4_⋅2H_2_O, marked as BM (567.21 g/mol). Syntheses of the V and B complexes were described by Przybylski et al. [21] and Krośniak et al. [22], respectively. The process of synthesis for the BM complex was similar to the one of the B complex, except for the molar ratio of ligand to vanadium, which was 2:1. The purity of all analyzed complexes was confirmed by microanalysis and IR spectroscopy. The chemical structures of the vanadium complexes are presented in Figure 1.

### 2.3. Animals

A total of 48 male Wistar rats, aged 3 months and weighing 250 ± 15 g, were divided into 8 groups. Each group of animals was fed with a different diet: control diet (group CN: 62% starch, 20% casein, 5.0% oil, 2.8% calcium carbonate, 2.9% Ca_3_(PO_4_)_2_, 1.0% lecithin, 0.3% NaCl, 4.7% cellulose, 1.0% minerals and vitamins mix, 0.07% MgO, and 0.23% K_2_SO_4_); control diet with the vanadium complex V—(2,2′-bipyridine) oxidobisperoxovanadate (V) octahydrate—(group CV); control diet with the vanadium complex B–bis (2,2′-bipyridine)oxidovanadium (IV) sulfate dehydrate—(group CB); control diet with the vanadium complex BM—bis(4,4′-dimethyl-2,2′-bipyridine)oxidovanadium (IV) sulfate dehydrate—(group CBM); high-fat diet (group FN: 32% starch, 20% casein, 5.0% oil, 30% lard, 2.8% calcium carbonate, 2.9% Ca_3_(PO_4_)_2_, 1.0% lecithin, 0.3% NaCl, 4.7% cellulose, 1.0% minerals and vitamins mix, 0.07% MgO, and0.23% K_2_SO_4_); high-fat diet with the vanadium complex V (group FV); high-fat diet with the vanadium complex B (group FB); and high-fat diet with the vanadium complex BM (group FBM). The tested complexes of vanadium were administered once a day for 5 weeks in a dose of 20 mg/kg of body mass. The rats were kept in a room with a constant temperature of 23 °C, 50–60% humidity, and a 12 h day-night cycle. The animals were euthanized by an intraperitoneal injection of sodium thiopental in a dose of 50 mg/kg of body mass. On each day of the experiment, the animals were weighed. On this basis, the necessary amount of test compounds was prepared, suspended in methylcellulose, and administered intragastrically by gavage.

### 2.4. Sample Collection and Analysis

The samples of adipose tissue were taken from visceral parts and were minced in 0.15 M phosphate buffer with pH 7.4 to 10% final concentration using an homogenizer Ultra-Turrax T25 ultra-speed tissue grinder (1200 r/min bursts) (IKA, Warszawa, Poland). All procedures were performed on ice. Homogenized tissues were centrifuged at 3000× *g* for 15 min (at temperatures up to 4 °C). From each obtained sample, a liquid layer was pipetted from under the fat layer, put into an Eppendorf tube, and frozen at −80 °C until the time of analysis [22].

### 2.5. Measurement of the Vanadium Content in Adipose Tissue

The analysis was performed on 111 ± 10 mg adipose tissue samples obtained from each tested animal group. The samples were treated with 2 mL of spectrally pure concentrated nitric acid (65%). The samples were incubated for 72 h and then diluted to a total volume of 3 mL. The measurement of vanadium was conducted using a 5100 ZL graphite furnace atomic absorption spectrometer with Zeeman correction and a L’vov platform (Perkin Elmer, Norwalk, CO, USA).

### 2.6. Relative Change of Rat Weight

The relative change in rat weight (∆*rw* [%]) was calculated as follows:

(1) $$\Delta rw\;\left[\%\right]=\frac{m_{33}-m_{3}}{m_{3}}\cdot 100$$

where *m*_33_ is the weight of the rat on day 33 of the experiment [g], and *m*_3_ is the weight of the rat on day 3 of the experiment [g].

### 2.7. Basal Metabolic Rate

The experiment was conducted in the following way: rats (*n* = 6) from a given group were divided into two subgroups (of 3 animals). For every subgroup, the total amount of feed and the total increase in body weight were calculated. In order to calculate how many grams of feed were needed for a 1 g increase in the animals’ body weight, a basal metabolic rate indicator was used. This indicator was calculated in the following way: the total feed consumption of the rats was divided by the total weight gain of the animals. The obtained result was then recalculated to the caloric demand for the animals’ body weight increase by 1 g, taking into consideration the applied diet (1 g of protein equals 4 kcal, 1 g of fat is 9 kcal, and 1 g of carbohydrates is 4 kcal). For the control diet, we calculated that 1 g of feed was equal to 3.8 kcal, and, in turn, 1 g of feed for the high-fat diet was 5.8 kcal. The results obtained for the tested animals from both subgroups were then averaged.

### 2.8. Measurement of the Total Antioxidant Capacity—Ferric Reducing Antioxidant Power (FRAP)

The Ferric Reducing Antioxidant Power (FRAP) assay, which was a modification of the Benzie and Strain method [23], was applied to measure the ability to reduce Fe^3+^ to Fe^2+^ ions in an acidic environment (pH 3.6). The absorbance was read at a wavelength of 593 nm after 30 min. The final results were expressed in mmol Fe^2+^/mg of protein. The protein content in all samples was determined by Bradford methods [24] using BSA as a control.

### 2.9. Measurement of Glutathione (GSH) Concentration

The concentration of glutathione (GSH) was measured according to the Ellman method [25]. The reduction of DTNB (5,5′-dithiobis(2-nitrobenzoic acid)) by thiol complexes was analyzed. The absorbance was read at a wavelength of 412 nm. The final results were expressed in nmol/mg of protein. Before the analysis of GSH, 2.5% trichloroacetic acid was used to remove interfering proteins from the samples. 

### 2.10. Measurement of Catalase (CAT) Activity

The activity of catalase (CAT; EC 1.11.1.6) was determined with the use of the kinetic method described by Aebi [26]. The measurements were performed spectrophotometrically at 240 nm, 25 °C. CAT concentrations were expressed in U/mg of protein. One unit of CAT activity was defined as the amount of enzyme decomposing 1 μmol of H_2_O_2_ per minute.

### 2.11. Measurement of Superoxide Dismutase (SOD) Activity

The Cu/Zn superoxide dismutase (E.C.1.15.1.1) enzyme activity was marked according to the Sun et al. [27], method. Superoxide dismutase (SOD) activity involves the inhibition of nitroblue tetrazolium reduction, with xanthine-xanthine oxidase used as a superoxide generator. The activity of this enzyme was marked based on the linear reading of absorbance changes between the fifteenth s and the second min at 550 nm wavelength, and it was expressed as U/mg of protein.

### 2.12. Statistical Analysis

All of the quantitative data are presented as the mean values ± standard deviation (food intake, energetic balance in animals, and concentration of vanadium element in adipose tissue). Two-way analysis of variance (ANOVA) was used to check if the analyzed parameters had influence on the variables. ANOVA was conducted for each of the following dependent variables: FRAP [mmol Fe^2+^/mg of protein], GSH [nmol/mg of protein], CAT [U/mg of protein], SOD [U/mg of protein], and ∆*rw* [%]. The type of diet (X1-diet) and the type of supplement (X2-supplement), in turn, were the intragroup factors. The type of diet (X1-diet) was analyzed on 2 levels (C and F), while the type of supplement (X2-supplement) was analyzed on 4 levels (N, B, V, and BM). In order to determine homogeneous groups (marked on graphs by the same letters), the least significant differences (LSD) of Fisher’s test were used. Data analysis was performed using Statistica (StatSoft, Inc. 2011 Inc., Tulsa, Oklahoma, USA). Values of *p* < 0.05 were considered statistically significant.

## 3. Results

Based on the results of previous research [28] in which the toxicity of the complex [VO (SO_4_) (2,2’-bpy)] 2H_2_O expressed as LD50 (mg/kg) was assessed, the animals were administered a dose of the tested complex in the amount of 20 mg/kg of body weight. The tested complexes were administered by gavage once a day for 5 weeks. Due to the lack of information on speciation in aqueous solution and on thermodynamic stability, the researched complexes were administered via an intragastric probe in the form of a methylcellulose mixture, which is a medium for distributing a weighed amount of complex compound.

Supplementation of each tested vanadium complexes, both in the control diet and in the high-fat diet, caused a statistically significant increase in its content in adipose tissue. No significant differences were observed between the individual vanadium complexes and the types of diet (Table 1). 

**Figure 2:**
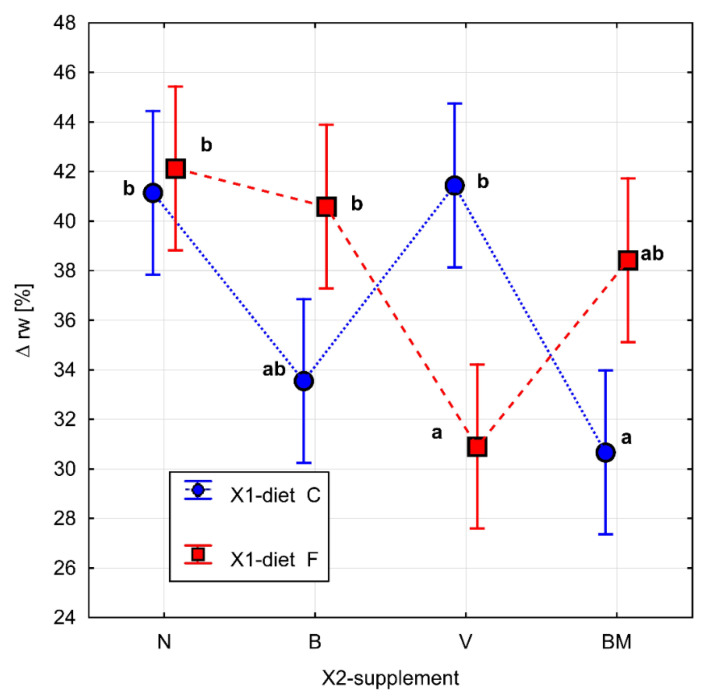
Relative change of rat weight: ∆*rw* (%). Diet (C: standard diet, or F: high-fat diet) without or with organic vanadium complexes (N: without organic vanadium complex; V: vanadium complexes; B: vanadium complexes; and BM: vanadium complexes). All data are expressed as means ± SEM, and *p* < 0.05 was accepted as statistically significant. Bars with a different letter are significantly different (*p* < 0.05).

**Figure 3:**
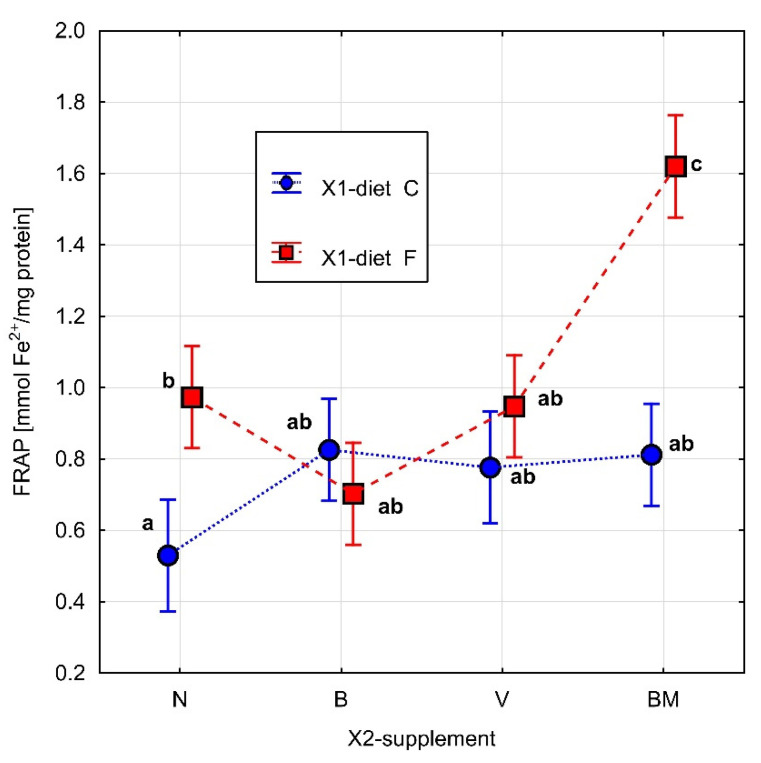
The total antioxidant capacity of homogenate of adipose tissue of rats is expressed as FRAP [mmol Fe^2+^/mg protein] after day 30. Diet (C: standard diet, and F: high-fat diet) without or with organic vanadium complexes (N: without organic vanadium complex; V: vanadium complexes; B: vanadium complexes; and BM: vanadium complexes). All data are expressed as means ± SEM, and *p* < 0.05 was accepted as statistically significant. Bars with a different letter are significantly different (*p* < 0.05).

**Figure 4:**
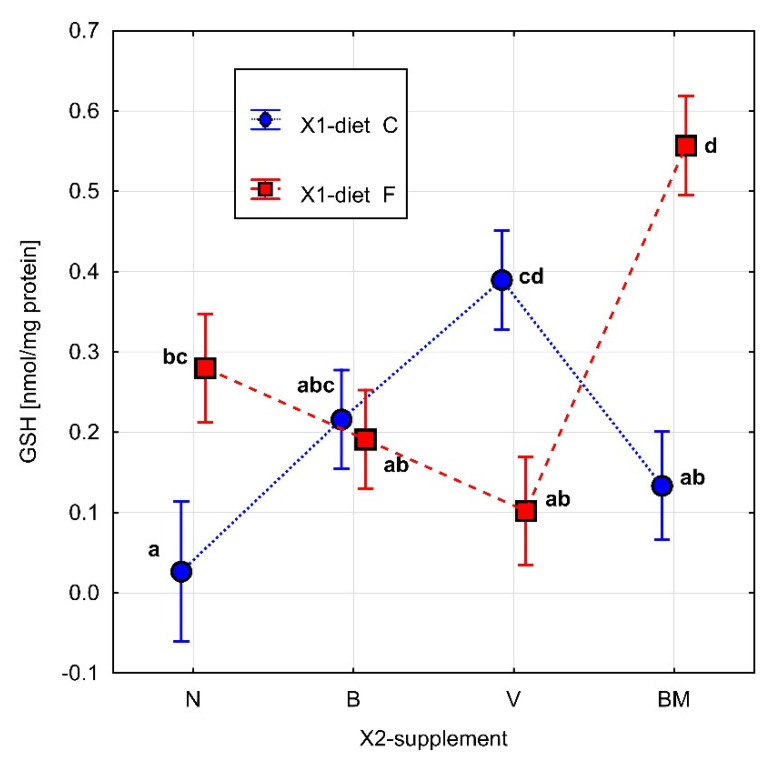
The concentration of GSH [nmol/mg protein] in homogenate of adipose tissue of rats. Diet (C: standard diet, and F: high-fat diet) without or with organic vanadium complexes (N: without organic vanadium complex; V: vanadium complexes; B: vanadium complexes; and BM: vanadium complexes). All data are expressed as means ± SEM, and *p* < 0.05 was accepted as statistically significant. Bars with a different letter are significantly different (*p* < 0.05).

**Figure 5:**
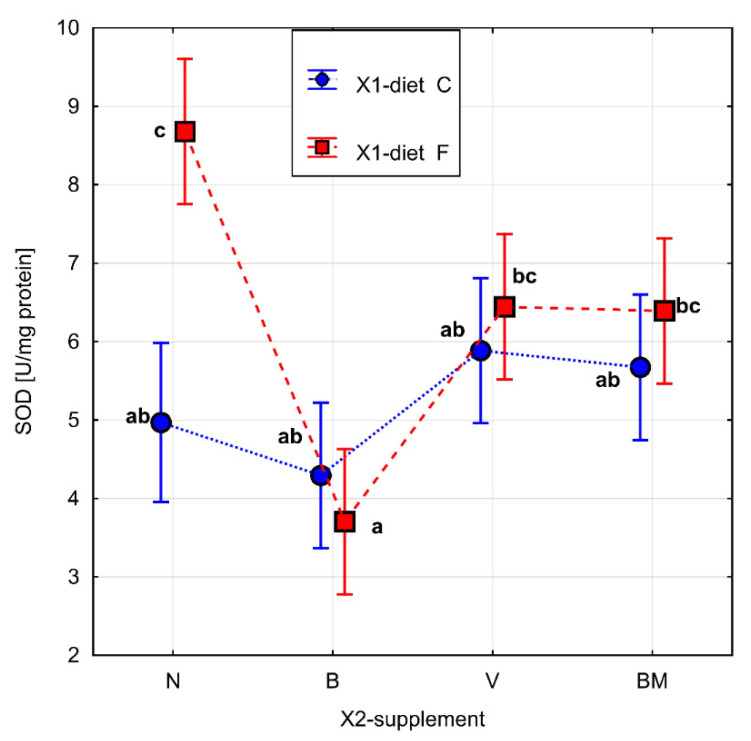
The activity of SOD [U/mg protein] in homogenate of adipose tissue of rats. Diet (C: standard diet, and F: high-fat diet) without or with organic vanadium complexes (N: without organic vanadium complex; V: vanadium complexes; B: vanadium complexes; and BM: vanadium complexes). All data are expressed as means ± SEM, and *p* < 0.05 was accepted as statistically significant. Bars with a different letter are significantly different (*p* < 0.05).

The parameters related to metabolic rate are presented in Table 1. The results of Fisher’s test for ∆*rw*, FRAP, GSH, and SOD are presented in Figure 2, Figure 3, Figure 4 and Figure 5, respectively. Homogeneous groups are marked with the same letters. Statistical analysis was performed for *p* < 0.05.

Based on two-way ANOVA, a statistically significant effect was demonstrated both for the applied diet model and for the tested vanadium complexes (*p* = 0.031) in the case of a relative change in rat weight (∆*rw*). The high-fat diet had a significant effect on the reduction of feed consumption compared to the control diet, regardless of the tested vanadium complexes.

The rats on the high-fat diet with the tested vanadium complexes (FV, FB, and FBM groups) consumed similar amounts of feed compared to the FN group (Table 1). Nevertheless, the effects of the vanadium complexes in combination with the high-fat diet on ∆*rw* were varied. In the group of rats fed with vanadium complexes and the high-fat diet (FV group), a significant decrease in the ∆*rw* value was observed. A similar effect on this parameter was observed in the group of rats on the control diet and BM complex (CBM group) (Figure 2). The rats in the control group consumed the same amount of feed, regardless of the supplemented vanadium complexes. Thus, the ∆*rw* reduction effect was due to the administration of the BM complex. The high-fat diet had a significant effect on reducing feed consumption compared to the control diet, regardless of the vanadium complexes.

The presence of the researched vanadium complexes in both types of diet significantly increased the basic metabolic rate and did not affect water intake (Table 1).

The types of diet (*p* = 0.003) and vanadium complex (*p* = 0.008), as well as the interaction between them (*p* = 0.017), had significant influences on FRAP (Figure 3). For the high-fat diet, the BM complex significantly increased the value of this parameter in a comparison of high-fat diet alone to the other tested complexes, i.e., V and B. Statistically significant differences were also observed between the types of diet. The high-fat diet caused a statistically significant increase in the FRAP value compared to the control diet. None of the tested vanadium complexes had a significant influence on the FRAP value in the control diet (Figure 3).

In the case of concentration of GSH, two-way ANOVA indicated a statistical effect only for the interaction of the tested diet with the vanadium complexes (*p* = 0.000). The BM complex in the high-fat diet induced a significant increase in GSH concentration in comparison to the other tested complexes, i.e., B and V, as well as in comparison to the high-fat diet (Figure 4). In the control diet, the V complex significantly increased the GSH concentration in comparison to both the BM complex and to the control diet (Figure 4). Statistical differences in the GSH concentration were also observed between the types of diet. The high-fat diet caused a statistically significant increase in the GSH concentration in comparison to the control diet. 

In the case of CAT activity, neither the tested factors–diet and vanadium complex–nor the interaction of these factors had a significant influence on the parameter (data not shown). 

The vanadium complex B (*p* = 0.027) had a significant effect on the activity of SOD in the high-fat diet. It significantly decreased the activity of SOD in comparison to the V and BM complexes, as well as to the high-fat diet itself. There were no significant changes in SOD activity in the groups of rats fed with the control diet without vanadium complexes or the control diet combined with the tested vanadium complexes (Figure 5). Statistical differences in SOD activity were also observed between the diet types without the addition of the tested complexes. The high-fat diet caused a statistically significant increase in SOD activity in comparison to the control diet.

## 4. Discussion

Vanadium complexes have been mainly studied as potential agents in the treatment of both types of diabetes [29,30,31,32,33,34]. Additionally, the vanadium (IV) complex called Metvan (where the ligand is 4,7-dimethyl-1,10-phenanthroline) has shown strong anticancer effects [35,36]. 

The results of various studies have indicated that an insufficient amount of vanadium in the diet may cause delayed growth and impaired reproduction or metabolism of lipids [37,38,39]. It was shown that organic vanadium complexes do not cause gastrointestinal complaints or liver and kidney toxicity [29]. However, in order to have clarity on the mechanisms of action for potential drugs based on organic vanadium complexes, it is important to understand how the complexes are absorbed and transported through the bloodstream to target tissues. 

For some V(IV) complexes that increase insulin levels, it was shown that solutions at pH 7.4 contain other forms of the complex than the solid state [40]. Sanna et al., investigated the method of connecting V(IV)O with bipyridine (bpy) ligands in aqueous solutions at various pH values, pointing to the role of the resulting cis-octahedral derivative forms at pH 7.4 in metal ion transport and mechanism of action [41]. The above-mentioned authors stated that the bis-chelated species of monomeric oxovanadium (IV) in solutions transformed into “the corresponding mono-hydroxido complex after the deprotonation of the water molecule“. This transformation occurred in solutions with pH 4.89.

The transformation of cis-[VO(bpy)_2_(H_2_O)]^2+^ into cis-[VO(bpy)_2_(OH)]^+^ was also presented by Triantafillou et al., who reported that the above-mentioned complexes were formed in an aqueous environment [42]. It was shown that the resulting form of cis could interact with bioligands and red blood cells. The speciation of the Metvan complex depends on the concentration of vanadium in blood [43]. At low concentrations (10 µM), its migration to the cell takes place in the form of the V(IV)O^2+^ ion, which is partially converted to V(V)O/V(V)O_2_.

The role of ligands in the cytotoxicity of V(IV) complexes was investigated by testing the stability of the complexes in neutral aqueous solutions and in cell culture media [44]. We noted the need to carefully consider the stability of the V(IV) and V(V) complexes in the media and the human toxicity of phen and bpy ligands. However, Levina et al., noted that the speciation of metal complexes critically depended on the nature of the medium in which the speciation was studied [45].

Tsave et al., conducted research using 3T3-L1 pre-adipocytes and did not confirm the cytotoxicity of organic ligands, such as 2-aminophenol, o-vanillin, 2-hydroxy-1-naphthaldehyde, salicylaldehyde, or 4,4′-bipyridine. [46]. Instead, they found that ligation increased the bioavailability of complexes and reduced the cytotoxic profile. However, the combination of ligands with V(V) showed in most cases a proliferative effect [46]. 

Several studies have shown that vanadium complexes with organic ligands are more active than inorganic salts and that they are not associated with adverse effects on the gastrointestinal tract [47,48]. Therefore, in our previous study, the influence of three organic vanadium complexes—sodium (2,2′-bipyridine)oxidobisperoxovanadate (V) octahydrate, bis(2,2′-bipyridine)oxidovanadium (IV) sulfate dehydrate, and bis(4,4′-dimethyl-2,2′-bipyridine)oxidovanadium (IV) sulfate dihydrate—on lipid metabolism under the condition of a high-fat diet in a non-diabetes animal model was analyzed [20]. In turn, our current research focused on the influence of the above-mentioned vanadium complexes on parameters related to metabolic rate, as well as selected antioxidant parameters in adipose tissue in the same animal model. 

Likewise, the studies by Zhang et al. [49] showed that vanadium complexes with concentrations below 20 µg/mL have low cytotoxicity. During an excessive supply of nutrients, the growth of white adipose tissue (WAT) prevents ectopic fat accumulation by affecting the rate of differentiation to adipocytes in preadipocytes [50]. Obesity has been shown to be closely related to adipocyte hyperplasia and the size of adipose tissue. Vanadium complexes in a dose-dependent manner can participate in the process of converting human preadipocytes into adipocytes and block the accumulation of cellular lipids, thus inhibiting the adipogenesis process, which, in turn, has an anti-obesity effect [49].

One of the hypotheses explaining the effect of vanadium complexes on the reduction of lipid concentration indicates the possibility of the formation of vanadium-protein complexes [49,51,52].

In obesity, there is increased oxidative stress in adipose tissue, as mitochondrial functions are impaired, which, in turn, leads to excessive production of reactive oxygen species (ROS) [53]. Obesity-related metabolic syndrome has its consequences, one of which is elevated oxidative stress in adipose tissue. Under these conditions, adipocytes, in which the production of adipocytokines may be disturbed, become the main source of increased levels of ROS, which leads to adipocyte dysfunction [50]. An increased ROS pool reduces the expression and secretion of adiponectin, an important protein for insulin sensitivity, which also exhibits anti-atherosclerotic and anti-inflammatory properties [54,55]. Under these conditions, adipocytes also show increased expression of NADPH oxidase and decreased expression of antioxidant enzymes, such as catalase, glutathione peroxidase, and superoxide dismutase [8,50].

Additionally, high-fat diets are involved in the induction of oxidative stress in adipose tissue as a key factor in the development of obesity [56,57]. Obesity, in turn, is related to systemic oxidative stress [58]. That is why some authors see the necessity of searching for new therapies enhancing the redox state in adipocytes [8]. Moreover, Tinkov et al. [59], put forward the hypothesis that the balance of some trace elements, such as vanadium, chromium, or zinc, in adipose tissue might be altered by caloric excess. Consequently, a decreased level of such minerals in that tissue might have deleterious effects on, inter alia, insulin resistance induction or proinflammatory adipocytokines production [59]. Therefore, research on the influence of vanadium complexes on antioxidative parameters in white adipose tissue under the condition of a high-fat diet seems to be well grounded. To the best of our knowledge, this issue does not seem to have been discussed so far.

In our study, the BM complex in the high-fat diet (FBM) significantly increased the activity of FRAP and the concentration of GSH in comparison to the high-fat diet (FN). The B complex in the same type of diet (FB) significantly reduced the activity of SOD in comparison to the FN, FV, and FBM groups.

Pillai et al. [15], observed the anti-oxidative properties of the vanadium-3-hydroxyflavone complex (vanadium on IV oxidation state, as well) in streptozotocin-diabetic rats. They demonstrated that the tested complex induced an increase in antioxidative parameters in the pancreas and in plasma. The activity of antioxidative enzymes (CAT, SOD, and GPx) was measured in the pancreas, while the concentration of GSH—a non-enzymatic antioxidant—was measured both in the pancreas and in plasma [15]. Liu et al. [48], also researched the influence of IV oxidation state vanadium complexes (oxovanadium (IV)/chitosan nanocomposites and the vanadyl (IV)–ascorbate complex) combined with high-fat and high-sucrose diets on the oxidative stress in liver tissue. The vanadium complexes analyzed significantly increased the antioxidant status in the liver tissue [47,48]. Xie et al. [60], also noted the influence of an organic vanadium complex (a vanadium-chlorodipicolinate compound) on changes in the activity of selected antioxidative parameters in the livers of streptozotocin-diabetic rats. This effect was dependent on the oxidation level of the vanadium in the complex. The vanadium (V)-chlorodipicolinate compound caused a statistically significant increase in both CAT and GPx activity in comparison to an animal group with diabetes. Simultaneously, the values were similar to those noted in the control group. A similar effect was also noted for the vanadium (IV)-chlorodipicolinate compound regarding GPx activity. In turn, the vanadium (III)-chlorodipicolinate compound had no effect on the values of these parameters. Regarding SOD activity, no influence of the three complexes was observed [60]. In our research, an increase of certain antioxidative parameters (FRAP and GSH) was seen in the case of the BM complex in a high-fat diet model. It should be underlined that in the cited works of the other authors the measurements of antioxidative parameters were performed in other tissues than in our research (i.e., in the pancreas, liver, and plasma). While the research model of those authors used also IV or V oxidation state vanadium complexes, the ligands linked with vanadium were different in comparison with ours. In the work of Pillai et al. [15], organic vanadium complexes containing flavone derivatives were used; Liu et al. [48], used polysaccharide ligands or L-ascorbic acid derivatives; and Xie et al., used chlorodipicolinate [60].

In our research, bipyridine (V and B complexes) or dimethylbipyridine (BM complex) were used as ligands; therefore, it is difficult to directly compare observations from our research to the results of other authors. Using MetaSite v 6.0.3, computer software Molecular Discovery (which mimics the natural metabolisms of compounds in different tissues, including liver tissue), changes in ligand lipophilicity in the vanadium complexes were analyzed for metabolism occurring in cytochrome P450 isoforms. Metabolic transformation of bipyridine (logP 1.72, Figure 1) leads to a change in compound lipophilicity as a result of aromatic hydroxylation that produces (4′–methyl [2,2′–bipyridin]–5–yl)methanol (LogP 1.37, Figure 1) or aliphatic carbonylation (4′–methyl[2,2′–bipyridine]–5)carbaldehyde (logP 1.86, Figure 1) and aliphatic carboxylation (4′–methyl[2,2′–bipyridine]–5)carboxylic acid (logP 2.05, Figure 1).

Our observations concerning the influence of vanadium in the BM complex on enhancing antioxidative status in adipocytes for a high-fat diet seem extremely interesting when compared to the results of Zhang et al. [51]. These authors suggested that vanadium (IV) in the form of vanadium (IV)-chlorodipicolinate plays a role in inhibiting processes of differentiation and adipogenesis in preadipocytes by lowering the expression or activity of adipogenic transcriptional factors and their target genes [51].

In the case of CAT, no differences were observed between the two types of diets (i.e., high-fat and control), which is a surprising observation. Interestingly, Sohet et al. [14] noted a statistically significant decrease in TBARS value (lipid peroxidase marker) in the visceral adipose tissue of a group of mice fed on a high-fat diet with 21% fructose in the drinking water compared to the control group in an 8-week experiment (3 weeks longer than ours), which may prove the complexity of the mechanisms responsible for maintaining oxido-reduction balance in adipose tissue when exposed to an excessive supply of calories. The only significant change observed in the case of the tested vanadium complexes combined with the control diet was an increase in GSH concentration in the case of supplementing with the V complex; the other tested complexes did not show any significant influence.

The type of diet may be a factor affecting vanadium content in adipose tissue [61]. Tinkov et al., noted that a high-fat diet induced a decrease in the content of vanadium in the fat tissue of Wistar rats by about 33% compared to control diet [62]. In turn, in our research, we noted a decrease in vanadium content in adipose tissue for the high-fat diet by only 4% in comparison to control diet. Regardless of the diet model (control or high-fat), a significant decrease in vanadium content in adipose tissue was noted.

It should be stressed that our research has some limitations, including a lack of inflammation markers in rat adipocytes, which undoubtedly would allow a broader overview of changes in adipose tissue for a high-fat diet and in the presence of the tested organic vanadium complexes. Furthermore, the lowest level of toxicity (LD50) of the newly synthesized vanadium complexes was not determined. The rating of the metabolic processes of individual ligands in the tested complexes was carried out solely in silico.

Based on our own experiments and other cited research results, vanadium seems to be an interesting alternative in the prevention and mitigation of obesity-related disorders, and, possibly, it will find a practical application in the future. However, continued research is indispensable in order to verify the above assumptions. Particular attention in future research should be paid to the BM complex. In our previous research, the BM complex in a high-fat diet had a statistically significant influence on both the increase in HDL concentration and total cholesterol in Wistar rat blood plasma in comparison to a high-fat diet [20]. These results, combined with observations from our current research, position the BM complex as a particularly interesting factor that may help reduce the negative health effects of an excessive supply of fats.

## 5. Conclusions

To summarize, among the tested organic derivatives of vanadium, the V and BM complexes significantly decreased the relative change in weight, which is particularly interesting in the context of the current overweight and obesity problem.

High-fat diets cause imbalance between the processes of reduction and oxidation in adipocytes. The BM complex combined with a high-fat diet had a beneficial effect on the total antioxidant capacity of adipocytes, as well as on the non-enzymatic parameter glutathione, influencing an increase in its concentration. The V complex combined with the control diet also had an impact on antioxidant status by increasing the concentration of glutathione, which might be important in the prevention of disturbance in the redox status of fatty tissue. Nevertheless, molecular research is necessary to understand the mechanisms of the interactions between the tested vanadium complexes and antioxidant status or energetic balance. It would also be interesting to examine properties of these complexes in other dietary models.

## Figures and Tables

**Table 1:** Food intake and energetic balance in the animals, and the concentration of vanadium elements in adipose tissue.

Animal Group	Water Intake ± SD [mL/Day/Animal]	Feed Intake ± SD [g/Day/Animal]	BWG ± SD [g/Animal]	BMR ± SD [kcal/g Body Weight Gain]	VI ± SD [mg/kg/day]	VCAT ± SD [μg/kg of Dry Adipose Tissue]
CN	27.0 ± 4.9 ^a^	18.4 ± 4.9 ^b^	105.3 ± 13.9 ^a^	21.0 ± 0.3 ^a^	ND	0.81 ± 0.09 ^a^
CV	30.3 ± 5.2 ^a^	17.3 ± 1.9 ^b^	96.8 ± 18.3 ^a^	22.6 ± 0.2 ^b^	2.25 ± 0.2 ^a^	3.48 ± 0.37 ^b^
CB	26.6 ± 2.5 ^a^	17.7 ± 3.6 ^b^	83.5 ± 15.4 ^ab^	26.1 ± 0.6 ^c^	1.99 ± 0.2 ^a^	3.67 ± 0.22 ^b^
CBM	28.0 ± 5.2 ^a^	17.9 ± 1.5 ^b^	76.8 ± 15.1 ^b^	27.6 ± 0.1 ^c^	1.80 ± 0.2 ^a^	4.02 ± 0.31 ^b^
FN	25.6 ± 3.6 ^a^	13.5 ± 0.8 ^a^	109.7 ± 21.5 ^a^	19.8 ± 1.2 ^a^	ND	0.78 ± 0.12 ^a^
FV	24.5 ± 3.8 ^a^	11.9 ± 2.2 ^a^	78.3 ± 14.1 ^b^	24.7 ± 1.0 ^b^	2.25 ± 0.2 ^a^	3.55 ± 0.29 ^b^
FB	29.2 ± 8.1 ^a^	12.3 ± 2.4 ^a^	95.6 ± 21.1 ^a^	23.1 ± 2.9 ^b^	1.99 ± 0.2 ^a^	3.53 ± 0.31 ^b^
FBM	29.6 ± 6.6 ^a^	13.3 ± 2.4 ^a^	94.5 ± 24.6 ^a^	24.6 ± 1.0 ^b^	1.80 ± 0.2 ^a^	4.19 ± 0.30 ^b^

BWG: body weight gain; BMR: basal metabolic rate; VI: vanadium intake; VCAT: vanadium concentration in adipose tissue; CN: standard diet without additives; CV: V vanadium complexes with standard diet; CB: B vanadium complexes with standard diet; CBM: BM vanadium complexes with standard diet; FN: high-fat diet without additives; FV: V vanadium complexes with high-fat diet; FB: B vanadium complexes with high-fat diet; and FBM: BM vanadium complexes with high-fat diet. Bars with a different letter are significantly different (*p* < 0.05), and ND means “not detected”.

## Data Availability

The data presented in this study are available upon request from the corresponding author.

## Abbreviations

V complex	sodium (2,2′-bipyridine)oxidobisperoxovanadate (V) octahydrate
B complex	bis(2,2′-bipyridine)oxidovanadium(IV) sulfate dehydrate
BM complex	bis(4,4′-dimethyl-2,2′-bipyridine)oxidovanadium (IV) sulfate dehydrate
CN	standard diet without additives
CV	V complex with standard diet
CB	B complex with standard diet
CBM	BM complex with standard diet
FN	high-fat diet without additives
FV	V complex with high-fat diet
FB	B complex with high-fat diet
FBM	BM complex with high-fat diet
∆*rw*	relative change in rat weight
